# *Asparagopsis* Genus: What We Really Know About Its Biological Activities and Chemical Composition †

**DOI:** 10.3390/molecules27061787

**Published:** 2022-03-09

**Authors:** José M. S. Ponte, Ana M. L. Seca, Maria Carmo Barreto

**Affiliations:** 1 Faculty of Sciences and Technology, University of the Azores, Rua Mãe de Deus, 9501-321 Ponta Delgada, Portugal; jmsponte@outlook.pt; 2 cE3c-Centre for Ecology Evolution and Environmental Changes/Azorean Biodiversity Group, Rua Mãe de Deus, 9501-321 Ponta Delgada, Portugal; ana.ml.seca@uac.pt; 3 LAQV-REQUIMTE, University of Aveiro, Campus de Santiago, 3810-193 Aveiro, Portugal

**Keywords:** *Asparagopsis*, *Asparagopsis armata*, *Asparagopsis taxiformis*, biological activities, antimicrobial, secondary metabolites, sterols, halogenated compounds

## Abstract

Although the genus *Asparagopsis* includes only two taxonomically accepted species, the published literature is unanimous about the invasive nature of this genus in different regions of the globe, and about the availability of large amounts of biomass for which it is important to find a commercial application. This review shows that extracts from *Asparagospsis* species have already been evaluated for antioxidant, antibacterial, antifungal, antiviral, antifouling, cytotoxic, antimethanogenic and enzyme-inhibitory activity. However, the tables presented herein show, with few exceptions, that the activity level displayed is generally low when compared with positive controls. Studies involving pure compounds being identified in *Asparagopsis* species are rare. The chemical compositions of most of the evaluated extracts are unknown. At best, the families of the compounds present are suggested. This review also shows that the volatile halogenated compounds, fatty acids and sterols that are biosynthesized by the *Asparagopsis* species are relatively well known. Many other non-volatile metabolites (halogen compounds, flavonoids, other phenolic compounds) seem to be produced by these species, but their chemical structures and properties haven’been investigated. This shows how much remains to be investigated regarding the secondary-metabolite composition of these species, suggesting further studies following more targeted methodologies.

## 1. Marine Algae as a Source of Bioactive Compounds

Oceans cover over 70% of the Earth’s surface. It is also estimated that more than 95% of all life on Earth inhabits the sea, which is populated by up to 100 million distinct species. The oceans, however, still hold many mysteries, as experts suggest that only about 5% of all marine biodiversity is currently known [1].

The sea is quite possibly Earth’s most valuable natural resource. Given its phenomenal biodiversity, the sea world is a vastly plentiful source of many different bioactive compounds. Marine organisms live in complex habitats and are often at the mercy of extreme environmental conditions. To defend themselves from predators, competitors and many other threats, sea creatures were forced to evolve chemical defenses. As would be expected, this has made it possible to discover a vast number of bioactive compounds produced by sea organisms. Many of these compounds cannot be found anywhere but the sea [1,2].

Over the course of several million years, marine algae have evolved many adaptations to marine life, including the production of several secondary metabolites. For this reason, and considering the huge diversity of marine algae, these organisms represent a very important source of useful compounds [3].

However, the biotechnological potential of algae and their usefulness to mankind is not limited to their secondary metabolites. Algae have been used as food for millennia, as is evidenced by 14,000-year-old archaeological findings in Chile [4] and early written accounts [5]. Some records suggest that algae such as *Eisenia* spp. and *Sargassum* spp. were used as food by the Japanese around 6000 B.C. Others account for the eating of 21 species of red, green, and brown algae in tenth century Japan [6]. Algae are also used in animal feed and as fertilizers [7]. Other applications include the use of algae as biofilters in wastewater treatment [7,8,9] and as energy sources, as algal biomass can be used as a substrate for biogas production by bacteria or for microbial fuel cells [7,10]. Algae are also used for biodiesel production [11] and are even being investigated as photobioreactors that could one day be part of the life-support systems of astronauts who are stationed at space stations or sent on long space journeys [12].

Several activities have been reported in scientific literature for both extracts and compounds isolated from several species of algae. Many of these compounds have human health benefits and commercial interest. For example, those that help prevent cardiovascular disease [13], such as the tripeptides IPP and VPP, which are used as bioactive agents in the commercial products Ameal-S 120^®^ from Japan and Evolus^®^ from Finland to control blood pressure [14,15]. Another example is the product Invincity^®^, which incorporates sulphated polysaccharides with iodine and bromine groups extracted from *Asparagopsis armata* as an anti-acne agent [16]. Several other compounds extracted from macroalgae that exhibit great pharmaceutical and industrial potential are detailed in recent reviews [17,18]. Many algal extracts that have been tested over the years have also displayed pharmacological properties, such as antioxidant activity [19,20], anti-inflammatory activity [19], antimicrobial activity [21], antiviral activity [22], anticancer activity [23], antiprotozoal activity [24] neuroprotective activity [25], among others. The cosmetics industry also benefits greatly from algal products, such as moisturizing ingredients [26], antioxidants [27], matrix-metalloproteinase inhibitors [28], photoprotective ingredients [29], and skin-bleaching ingredients [30], among others. Some of the above-mentioned activities will be further detailed in later sections.

## 2. The Genus *Asparagopsis*

According to the AlgaeBase database [31], the genus *Asparagopsis*, belonging to the order Bonnemaisoniales of the phylum Rhodophyta (red algae), includes three taxonomically accepted species: *Asparagopsis armata* Harvey, *Asparagopsis svedelii* Taylor and *Asparagopsis taxiformis* Trevisan. However, opinions regarding the taxonomic validity of the species can vary depending on the author [32]. Taylor [33] described *A. svedelii* from the Galapagos islands. However, *A. svedelii* has not been referenced in the scientific literature since its original description. Gland cells and reproductive structures were not described, and this seaweed seems to more closely resemble *Bonnemaisonia hamifera* rather than other *Asparagopsis* species, which has led some researchers to question its taxonomic validity [34].

One reason for the researchers’ raised curiosity about the genus *Asparagopsis* is the fact that *A. armata* was the first red alga in which a heteromorphic life cycle was observed [35]. This life cycle includes the filamentous “genus” *Falkenbergia*. *A. armata* carpospores germinate to produce a filamentous, free-floating, diploid and asexual tetrasporophyte that was identified as *Falkenbergia rufolanosa*, based on *Polysiphonia rufolanosa* [35,36]. During this tetrasporophyte stage, *A. armata* is brownish red in color, and consists of highly branched, filamentous, cotton-wool-like tufts that grow up to 15 mm in diameter. This stage can be found all year round but is more evident from October to March in Western Europe. The haploid-gametophyte stage occurs from June to September, and has a plant-like structure, up to 20 cm in length, with barbed branches and a rosy/yellowish-pink color that becomes reddish after the specimens have been removed from sea water [31]. Chihara [37] observed that *A. taxiformis* has the same life cycle as *A. armata* and identified the tetrasporophyte as *Falkenbergia hillebrandii*, based on *Polysiphonia hillebrandii*. However, experts believe that the *Falkenbergia* stages of both species are indistinguishable [36,38]. A review by Zanolla et al. [39] concisely summarizes what is known about the genetics, phylogeny and phylogeography of the *Asparagopsis* genus. Preliminary work on the genetic characterization of *Asparagopsis* spp. was done by Chualaín et al. [32], who found that the analysis of small subunit rRNA sequences and restriction-fragment-length polymorphisms (RFLPs) allowed distinction between *A. armata* and *A. taxiformis*, and that RFLP analysis also allowed the distinction of two clades of *A. taxiformis*, one found in tropical locations and another in temperate locations. More on this topic can be found in the review by Zanolla et al. [39], which highlights the fact that, despite comprising very few species, the genus *Asparagopsis* displays remarkable genetic diversity.

*A. taxiformis* and *A. armata* have different geographical distributions. *A. taxiformis* is widely distributed in tropical and subtropical regions [34,40]. *A. armata*, on the other hand, seems to be native to the southern hemisphere [32] and was introduced to the Atlantic and the Mediterranean in the 1920s [36], probably imported from southern Australia where it has been found in abundance for a long time [31,41,42]. Both gametophytes and tetrasporophytes were virtually simultaneously found in four distinct European locations, which is thought to be the result of four different introduction events [43,44]. A few preliminary ecophysiological studies seem to support this hypothesis [45]. The *Falkenbergia* stage quickly spread across the British Isles [46,47] and *A. armata* is now widely distributed from the North Atlantic to the coast of Senegal [48] and in the Mediterranean basin [49].

In the Mediterranean, the genus *Asparagopsis* is considered one of the worst biological invaders and is a significant threat to the local biodiversity. The free-floating tetrasporophyte and the gametophyte with the ability to attach itself to floating structures and to propagate through fragmentation are crucial to this genus’ invasive success [50]. These algae form monospecific coverages and dominate many algal assemblages [51,52,53,54]. Competition for resources, especially space and food, is one of the main causes of displacement of native species by invading ones. This behavior has been observed in several macroalgae, such as *Asparagopsis* spp., which are more efficient in competing for resources than native microalgae and sessile invertebrates, forcing the latter to be displaced and reducing the habitat’s species richness [55,56]. The fact that the tetrasporophyte stage has a large surface-to-volume ratio is one of the reasons why it has more potential for rapid nutrient intake than other algae, contributing to the depletion of available nutrients [57,58,59]. *Asparagopsis* monospecific coverages can be so thick that they can reduce the amount of sunlight available for other primary producers, including native photosynthetic species [60]. *A. armata* has been seen covering 100% of the upper infralittoral area (depths of 0–10 m) during the winter in the northwest of the Mediterranean [61]. These high-density populations are probably sustained by the fact that some of the Mediterranean’s main herbivores (e.g., the sea urchin *Paracentrotus lividus* and the perciform fish *Sarpa salpa*, commonly dreamfish or salema) avoid feeding on *A. armata* [52]. Aside from the direct impact on biodiversity, there is also evidence suggesting that *Asparagopsis* spp. have an indirect economic impact, negatively affecting fishing and aquaculture, mainly through fixation to fishing nets and submerged aquaculture structures (e.g., cages and ropes), as well as sea-related touristic and recreational activities [53,60].

Despite its negative impact, algae of the genus *Asparagopsis* do display a great deal of biotechnological potential. Perhaps highlighting this potential, and the possible profits involved, could make it more tempting for authorities to act regarding the negative impacts of the invading algae. Indeed, these algae are directly explored by mankind. *A. taxiformis* has cultural significance and has been used as food by Hawaiians for a long time [62] and *A. armata* is commercially farmed in northern Europe in order to extract bioactive molecules [16]. The potential of *Asparagopsis* spp. as a seaweed biofilter for the effluents of commercial fish farms, for example, has been demonstrated by Schuenhoff et al. [8]. It has also been shown that the addition of *Asparagopsis* spp. into the diets of bovine cattle could potentially reduce methane emissions [63], methane being a gas that has about 25 times the global-warming potential of carbon dioxide. Zhu et al. [64] published a comprehensive review of the anti-methanogenic activity of *Asparagopsis* spp., in which the authors discuss the potential of *Asparagopsis* spp. to reduce methane emissions from ruminants, focusing on the mechanism of methane production by ruminants, the proposed pathways for halocarbon biosynthesis by *Asparagopsis* spp. and on the status and limitations of scale production of *Asparagopsis* spp. biomass. The genus *Asparagopsis* is also an immense source of natural halogenated compounds. Bromoform (CHBr_3_) is the most abundant of more than 100 compounds containing bromine, chlorine and iodine, acetic and acrylic acids and cyclic compounds that have been isolated from species of this genus [65,66,67,68,69]. These and other compounds, as well as extracts of *Asparagopsis* spp. have exhibited several biological activities, some of which will be discussed in further detail in the following sections.

## 3. Biological Activities

### 3.1. Antioxidant Activity

The biochemical reactions that continuously occur inside every single living cell are the driving force that sustains life. Yet many of these reactions generate free radicals, i.e., atoms, molecules or ions with unpaired electrons that are highly unstable and reactive [70]. Several internal (e.g., mitochondrial activity, peroxisomes, etc.) and external factors (e.g., tobacco smoke, radiation, solvents, etc.) stimulate the generation of free radicals [71]. The balance between the generation of free radicals and their neutralization by endogenous antioxidants is quite delicate. If the scale tips in favor of an excessive number of free radicals, cells will start to suffer the effects of oxidative stress, which can include severe cellular damage (primarily to DNA, proteins and lipids) and even death [72].

Damage caused by free radicals has been associated with several serious illnesses, such as cancer, cardiovascular diseases (e.g., hypertension, atherosclerosis, cerebrovascular accidents, vasculitis), neurological disorders (e.g., Parkinson’s, Alzheimer’s, Huntington’s, autism), renal disorders (e.g., glomerulonephritis), liver disorders, rheumatoid arthritis, adult respiratory-distress syndrome, auto-immune diseases (e.g., systemic lupus erythematosus), inflammation, cataracts, gastric ulcers, hemochromatosis, among others [72]. Several studies have also shown a link between oxidative stress and the aging process [73,74,75]. One of the most substantial causes of aging is the accumulation of functional damage on cells, particularly damage affecting DNA [71]. Oxidative stress, especially the stress caused by exposure to UV radiation, is also largely responsible for skin aging [73]. Given all the undesirable effects of free radicals, it follows that reducing those effects could help in the prevention of several serious illnesses and delay the aging process. Enter antioxidants.

As photosynthetic organisms, marine algae are exposed to a combination of intense light and high concentrations of oxygen that stimulates the generation of free radicals. The fact that marine algae show remarkable resistance to oxidative damage suggests that their cells possess highly effective antioxidant defenses [76]. Some of the antioxidant substances most frequently found in algae include polyphenolic compounds, vitamins and photosynthetic pigments [2].

Several studies conducted with *Asparagopsis* spp. have revealed that these algae produce compounds with antioxidant activity. The most significant results can be seen in Table 1.

Zubia et al. [77] tested the antioxidant activity of dichloromethane–methanol (1:1) extracts of several Rhodophyta from the coast of France. Among the tested species, *A. armata* extracts were among those that exhibited the highest free-radical-scavenging activity in the DPPH (2,2-diphenyl-1-picrylhydrazyl) assay. However, the obtained results (an EC_50_ of 6.25 mg/mL) indicate a weak antioxidant activity. A large concentration of extract, 104 to 446 times larger than the concentration of the positive controls used, was required in order to produce the same antioxidant effect. Using the same assay, Rhimou et al. [78] obtained better results for the methanol extract of *A. armata* from the Mediterranean Moroccan coast, but a concentration 78 to 107 times higher than that of the positive controls was still required in order to produce the same effect. Both Zubia et al. [77] and Rhimou et al. [78] also tested the antioxidant activity of *A. armata* extracts using the β-carotene/linoleic acid assay. Zubia et al. [77] verified that the extract of *A. armata* had inferior results when compared to the extracts of the other studied species, with a percentage of oxidation inhibition varying between 8.02% and 4.92% for concentrations varying between 50 and 500 μg/mL, respectively. Surprisingly, the percentage of inhibition in this assay seems to be inversely proportional to the concentration of the extract. This did not occur for any of the other tested species or the positive controls and seems to suggest that the *A. armata* extract could have pro-oxidant rather than antioxidant activity, which seems to go against the results of the DPPH assay. However, other factors could influence these results. The DPPH and β-carotene/linoleic-acid-assay test for different mechanisms of antioxidant action and the results of these two assays will not always correlate. Additionally, the β-carotene/linoleic-acid assay seems to be affected by many factors that limit its reproducibility. In a paper by Dawidowicz and Olszowy [83], the authors found that both the type of solvent and the volume of solvent used in the measuring system significantly influence the results of the assay. As an example, the researchers found that the volume of ethanol used was inversely proportional to the antioxidant activity of BHT. Rhimou et al. [78] also had very poor results for the methanol extracts of *A. armata* using the β-carotene/linoleic-acid assay. Looking at the results of these two studies, it seems that the methanol extract of *A. armata* contains a larger amount of antioxidant compounds than the dichloromethane–ethanol extract. However, there are significant differences for similar controls between the two studies, making it difficult to compare results. These differences could be the result of methodological differences between the two studies.

Neethu et al. [81] used several methods to test the antioxidant activity of chloroform, methanol, petroleum ether, and ethyl acetate extracts of *A. taxiformis*. In the hydrogen-peroxide-scavenging assay, all the extracts showed scavenging activity in a concentration-dependent manner. However, it was significantly lower than the activity observed for the positive control (ascorbic acid), which was surprisingly low itself, at 17.59 % inhibition at 500 µg/mL. Other examples found in literature [84,85] show that ascorbic acid is a far better scavenger of hydrogen peroxide. In the assay that tests the capacity for superoxide-radical scavenging, it was observed that the percentage of scavenging increases with the concentration of the extract. Among the tested extracts, methanol (85%) and chloroform (79%) exhibited highest scavenging activity at 500 μg/mL. The positive control (ascorbic acid) showed a similar scavenging effect (87%) to that of the methanol extract. The FRAP (ferric-reducing-antioxidant power) assay revealed maximum antioxidant activity for the chloroform extract (67%) at 100 μg/mL. The positive control (ascorbic acid) showed a similar capacity to reduce Fe^3+^ (73%) when compared to the extracts.

Nunes et al. [82] studied the antioxidant activity of *A. taxiformis* extracts as well as their ferrous-ion-chelating activity, which measures the ability of secondary antioxidants to inhibit oxidation through an indirect approach. The results can be seen in Table 1. These researchers used two different extraction methods (M1, by sonication and stirring, and M2, using Soxhlet extraction) to obtain each extract and found that there were significant discrepancies in the results for extracts that used the same solvent but different extraction methods. The ethanol extract obtained using M1 had the best results in both antioxidant-activity assays and was the extract with the largest amount of total phenolic compounds and chlorophyll a, which are compounds known to have antioxidant activity.

These studies suggest that algae of the genus *Asparagopsis* produce metabolites that exhibit antioxidant activity. Further research is required to isolate the active compounds and to determine their mode of action.

### 3.2. Cytotoxic Activity

Historically, cancer has always been, and still is, a major cause of human fatalities. Prospects are not reassuring either, since the number of cancer cases is expected to increase as populations grow larger, older and continue to adopt lifestyles that increase the risk of cancer [86]. It is estimated that, in 2020 alone, the number of new cancer cases worldwide was around 19.3 million and that 10 million people died of the disease [87].

In the last three decades, almost 80% of all cancer-fighting drugs approved by the FDA (Food and Drug Administration, USA) were natural products or synthetic products based on natural products [88] and 60% of all commercially available anticancer drugs were of natural origin [89].

There are not many published papers about the anticancer activity of *Asparagopsis* spp., but there is enough available information to suggest that these algae have some potential as producers of cytotoxic compounds. Zubia et al. [77] tested the cytotoxic activity of the dichloromethane–methanol (1:1) extracts of several Rhodophyta from the French coast against Daudi cells (in vitro model of Burkitt’s lymphoma), Jurkat cells (in vitro model of T-cell leukemia) and K562 cells (in vitro model of myeloid leukemia). The results for the *A. armata* extracts can be seen in Table 2. No positive control was used and the IC_50_ values were not calculated, making it difficult to draw any conclusions. However, a large concentration of extract seems to be required in order to produce a significant effect.

Alves et al. [89] tested the cytotoxic and antiproliferative activity of methanol and dichloromethane extracts of several algae from the Portuguese coast against an in vitro model of human hepatocellular carcinoma (HepG-2 cells). At a concentration of 1 mg/mL for 24 h, the methanol extract of *A. armata* was capable of reducing the percentage of viable cells to 11.22 ± 2.98 % of the control population. The dichloromethane extract further reduced that population to 1.51 ± 0.38%. The IC_50_ values can be seen in Table 2. While this is a very significant reduction in the percentage of viable cells, it is important to note that 1 mg/mL is a very high concentration, as are the IC_50_ values. The same is true in the antiproliferative assay, where a very large concentration of extract, 30 to 50 times larger than that of the positive control tamoxifen, was required in order to produce that same effect. Alves et al. [90] also found that methanol and dichloromethane extracts of *A. armata* have cytotoxic and antiproliferative activity against Caco-2 cells, an in vitro model of human colorectal cancer, and Nunes et al. [91] found that a chloroform–methanol (2:1) extract of *A. taxiformis* had some cytotoxic activity against A549 cells, an in vitro model of lung cancer cells.

These studies suggest that *Asparagopsis* spp. produce metabolites with some anticancer activity. However, a lot of research remains to be done in order to isolate, identify and purify the molecules that have anticancer potential and to understand the intracellular signaling processes that are associated with the cytotoxicity and/or cell cycle regulatory mechanisms [89].

### 3.3. Antimicrobial Activity

The appearance of bacterial strains that are resistant to multiple antibiotics, i.e., the multiresistance issue, has prompted researchers to look for new products with antimicrobial activity [92]. Since many marine organisms, including algae, produce bioactive metabolites as a response to ecological pressure, the exploration of this chemical diversity has led to the discovery of several compounds with antimicrobial activity [93]. Marine pathogenic bacteria can have a serious impact on algae [94,95,96]. In addition, epiphytic bacteria can negatively affect algae through the increase in hydrodynamic drag and the inhibition of photosynthesis due to fouling (i.e., the accumulation of microorganisms, plants or animals on the surface of submerged organisms or structures) [97]. Notwithstanding the constant threat of bacterial epibiosis, algae can keep themselves relatively free of bacterial diseases and excessive fouling [96,98]. These observations have spurred research on algae as a source of antimicrobial compounds [99].

Paul et al. [100] observed that dichloromethane and methanol extracts of *A. armata* exhibited antibacterial activity against *Vibrio* spp., *Escherichia coli*, *Pseudomonas aeruginosa* and *Staphylococcus* spp. and used gas chromatography and mass spectrometry to determine the composition of the algal extracts. Bromoform and dibromoacetic acid were the dominant compounds in the extracts of *A. armata*, and both these halogenated compounds were found to be capable of inhibiting the growth of all the tested bacterial strains. In this study, the researchers also found that antibacterial metabolites were produced in specialized cells and that *A. armata* possesses systems that allow the release of these metabolites into the surrounding seawater. By creating an artificial seawater medium deprived of bromine, the researchers found that the algae that were unable to produce halogenated compounds were colonized by a significantly larger number of epiphytic bacteria than those that did produce halogenated compounds. These results suggest that the ecological function of these metabolites is associated with defense against colonization by epiphytic bacteria.

Bansemir et al. [101] tested the activity of dichloromethane, methanol and water extracts of several algae against several fish pathogenic bacteria (*Aeromonas salmonicida* ssp. *salmonicida*, *Aeromonas hydrophila* ssp. *hydrophila*, *Pseudomonas anguilliseptica*, *Vibrio anguillarum*, *Yersinia ruckeri*). The results of the disk-diffusion assay can be seen in Table 3. Only the dichloromethane extracts exhibited significant antibacterial activity and *A. armata* was the alga that showed the strongest activity out of the tested species. As an example, the MIC (minimal inhibitory concentration, i.e., the lowest concentration of antibiotic that prevents bacterial growth) for the dichloromethane extracts of *A. armata* against *V. anguillarum* was <100 μg/mL. This value might be significantly larger than the MIC of the positive control oxytetracycline (0.5 μg/mL) but that is to be expected. While oxytetracycline was used in a purified state and at a known concentration, extracts are complex mixtures of several compounds, which means that whatever compounds are responsible for the antibacterial activity could be highly diluted. Bansemir et al. [101] highlighted the potential of *A. armata*’s bioactive compounds as prophylactics or therapeutic agents in the treatment of fish bacterial infections that could be used in aquaculture and fishkeeping. The inclusion of *A. armata* in fish diets could be an alternative to the application of extracts, fractions or purified compounds, according to the researchers. However, further research on the possible toxicity of the algae against fish and on the stability and metabolization of algal bioactive compounds is required in order to confirm whether these ideas can be employed in the aquaculture industry.

Mata et al. [109] investigated whether *A. taxiformis* has the potential to be integrated into fish aquaculture systems and to act as a therapeutic agent against fish pathogenic bacteria. Like *A. armata*, *A. taxiformis* is also capable of producing and releasing halogenated antibacterial metabolites into the surrounding seawater [109]. The authors quantified the release, accumulation, and residence of such metabolites in the cultivation medium and used this medium to test its in vitro activity against *Streptococcus iniae*, a common fish pathogen that is often a problem in aquaculture systems. The results suggest that the medium delayed but could not inhibit the growth of *S. iniae*, even though a treatment with concentrations of bromoform and dibromoacetic acid three orders of magnitude higher (70 mg/L and 32 mg/L, respectively) than that of the water that was directly sourced from an operational cultivation tank did inhibit the growth of *S. iniae* by more than 80%. However, increasing the concentration of metabolites becomes harmful to fish even at non-antibacterial concentrations. These results suggest that *A. taxiformis*’ metabolites have limited potential in the treatment of barramundi (*Lates calcarifer*) infected with *S. iniae*.

Salvador et al. [102] tested the activity of solid algal mass (i.e., obtained without solvents) and of methanol extracts of 82 Iberian macroalgae against three Gram-positive bacteria, two Gram-negative bacteria and one yeast. The results showed that the algae with the strongest antimicrobial activity belonged to the order Bonnemaisoniales, particularly *A. armata*, which was one of the algae that exhibited the strongest activity and the broadest spectrum (Table 3). Based on the observed activity, the researchers suggested the potential of algal metabolites as natural preservatives in cosmetics.

Pinteus et al. [93] tested the activity of methanol, *n*-hexane and dichloromethane extracts of twelve algae from the Portuguese coast against *Escherichia coli* (Gram-negative bacteria), *Bacillus subtilis* (Gram-positive bacteria) and *Saccharomyces cerevisiae* (yeast). Even though there were no positive results against *E. coli*, the extracts of *A. armata* exhibited the strongest activity against *B. subtilis*. The activity was significantly weaker than that of the positive control chloramphenicol. Nonetheless, that is to be expected since, as previously mentioned, extracts are complex mixtures in which the bioactive compounds can be present at very small concentrations. The *n*-hexane and dichloromethane extracts of *A. armata* were also capable of inhibiting the growth of *S. cerevisiae*, as can be seen in Table 3. The positive control amphotericin had a significantly better performance, but again the difference could be attributed to the diluted state of the bioactive metabolites present in the extracts.

Greff et al. [69] tested the antibacterial activity of two newly discovered brominated cyclopentones: mahorone and 5-bromomahorone. Both cyclopentones exhibited moderate antibacterial activity against the human pathogen *Acinetobacter baumannii*, as can be seen in Table 3, as well as the results against other bacteria and fungi. There was also some relevant activity against Methicillin-resistant *Staphylococcus aureus* (MRSA). The choice to use MIC_80_ as a metric for antibacterial activity, instead of the more common MIC or MIC_50_, is an odd one, and makes it hard to compare these results to those of other studies.

Besides the antibacterial activity per se, a study by Jha et al. [110] suggests that *Asparagopsis* spp. produce compounds that can interrupt quorum sensing. Quorum sensing is a population density-dependent gene-regulation system mediated by bacterial extracellular-signaling molecules that is used by bacteria to regulate the formation of biofilms (i.e., organized microbial communities that sheath themselves in extracellular polymeric substances that can confer antibiotic resistance to the bacteria) [111] and their pathogenicity and production of virulence factors [112]. Therefore, quorum sensing could be the ideal target for antipathogenic drugs that could be an alternative to antibiotics, which have led to the emergence of antibiotic resistance in bacteria [113]. It has been shown that quorum-sensing inhibitors can increase the susceptibility of biofilms to antibiotics both in vitro and in vivo [114]. Biofilms can have adverse effects on several important structures such as water-supplying pipes, air ducts and industrial fermenters. They cause erosion, clogging and the formation of slippery coatings on surfaces, as well as the accumulation of potentially harmful bacteria [115,116]. Marine biofilms (fouling) are one of the main causes of economic loss in maritime industries [117,118]. The most common strategy employed to fight fouling involves the application of paints and coatings that contain anti-fouling chemicals that prevent the accumulation of organisms on structures. These paints and coatings have been mostly based on heavy metals such as copper, chromium and tin. However, many studies have demonstrated that heavy metals have negative impacts on several marine microorganisms and mollusks [119,120]. Since marine algae keep themselves relatively free of fouling, it was suggested that, in addition to microbial compounds, marine algae could also produce quorum-sensing inhibitors. Indeed, the first quorum-sensing inhibitor was isolated from the red alga *Delisea pulchra* [121]. Jha et al. [110] observed that, in addition to inhibiting the growth of *Chromobacterium violaceum*, methanol extracts of *A. taxiformis* and some of their fractions also inhibited quorum sensing in *C. violaceum* and *Serratia marcescens*.

Pinteus et al. [106] investigated the ability of *A. armata* to inhibit the growth of several marine and freshwater bacteria and marine microalgae, in the context of finding greener alternatives to common antifouling solutions. The results can be seen in Table 3. The researchers also fractioned the crude methanol–dichloromethane (1:1) extract using vacuum liquid chromatography. The fractions were, generally, more effective than the crude extracts at inhibiting bacterial growth. This points out the importance of isolating the compounds that are responsible for this activity, as they are present in low concentrations in the crude extracts. The results also suggest that *A. armata* produces several products with broad-spectrum biocidal activity. The researchers also investigated the inhibition of bacterial biofilm formation, which is an essential step in the fouling process. All but two fractions and crude extracts were effective against biofilm formation by *Vibrio parahemolyticus*, with percentages of inhibition ranging from 13% to 30%, in concentrations ranging from 125 to 500 µg/mL. In the case of biofilm production by *B. subtilis*, the crude extract was the most effective, with the highest inhibitory activity ranging between 49% and 59%, with extract concentrations of 125 and 500 µg/mL, respectively. The extracts and fractions also showed inhibitory activity against the enzyme acetylcholinesterase, which is essential to the settling of invertebrates in a specific substrate, meaning that compounds produced by *A. armata* are capable of inhibiting fouling by acting on several key stages of the process, such as bacterial and algal growth, biofilm production and chemical signaling.

Activity against *Leptospira* spp., the bacteria responsible for the disease leptospirosis, has also been observed. Vedhagiri et al. [122] tested the antibacterial activity of methanol extracts of *A. taxiformis* against several strains and isolates of *Leptospira javanica* and calculated the MIC, MBC (minimum bactericidal concentration) and EC_50_ values, which ranged from 100–400 μg/mL, 200–1600 μg/mL and 34.48–322.9 μg/mL, respectively. The results for the extracts were often comparable to those of the positive controls penicillin and doxycycline.

Antiprotozoal activity against *Leishmania donovani*, one of the protozoans responsible for causing the disease leishmaniosis, has also been reported. Genovese et al. [107] found that extracts from both *A. armata* and *A. taxiformis* had a significant inhibitory effect *on L. donovani*. Results are in Table 3 and suggest that the active compounds are non-polar or moderately polar. LC-MS analysis indicates that brominated compounds are responsible for this activity.

Although several studies have demonstrated some degree of antimicrobial activity of extracts from *Asparagopsis* spp., most of these studies use the disk-diffusion method, or variations of it. This method is based on the inhibition of plated bacteria by antibiotics present in paper disks. The antibiotics diffuse through the agar medium and are present at progressively lower concentrations as the distance to the disk increases. If the antibiotic is effective, no bacteria will grow around the disk. The area around the disk where no bacterial growth is observed is referred to as the inhibition zone or inhibition halo. The larger the zone, the more sensitive the tested bacteria are to the tested antibiotics. However, even though the MIC of an antibiotic influences the size of the inhibition zone, this test cannot be used to determine the MIC value, mostly because the diameter of the inhibition zone is also a function of the initial antibiotic concentration and of its solubility and rate of diffusion in the agar. This means that the disk-diffusion method cannot be used to directly compare the effectiveness of two different antibiotics [123] and makes it hard to compare the results of studies that use this method. The method is, however, good enough to show that antimicrobial activity does take place. It is also important to standardize the units used to quantify activity, so that different studies can be compared.

Most research so far has been done on the activity of extracts and not pure compounds, which means that there is further work to be done to isolate active compounds and determine their mode of action.

### 3.4. Antiviral Activity

The last two years have been a harsh reminder of the dangers that viruses pose to mankind. At the time of writing, COVID-19, caused by the virus SARS-CoV-2, has claimed more than six million lives worldwide. Viruses are all around us. They are the most abundant “lifeforms” in the ocean [124], with an estimated 10^30^ viral particles existing in the oceans alone [125]. They exist in the atmosphere, with billions of viral particles depositing on a single square meter above the atmospheric boundary layer [126]. They lurk in the smallest bits of soil [127]. If the estimated 10^31^ individual virus existing on Earth were laid end to end, they would stretch for 100 million light years. It is also estimated that 10^23^ viral infections happen each second in the ocean [128]. Viruses are ubiquitous, have a remarkable ability to mutate [129], and have zoonotic capabilities. Indeed, most new infectious diseases have a zoonotic origin [130], in which the virus is capable of crossing the species divide and infecting humans. It is estimated that, in mammal and bird hosts alone, there are approximately 1.67 million undiscovered viral species in key zoonotic families, and experts claim that we can reasonably expect that between 631,000 and 827,000 of these unknown viruses have zoonotic potential [130]. With this in mind, we can safely assume that it is only a matter of time before the next viral outbreak strikes. Additionally, as COVID-19 revealed, we, as a species, are wildly unprepared to deal with a pandemic. Therefore, it is important to keep researching natural products to find compounds with antiviral activity.

At least three studies have demonstrated that extracts and compounds isolated from *Asparagopsis* spp. have antiviral potential. Shalaby and Shanab [131] tested the antiviral activity of *A. taxiformis* extracts and pure compounds against H5N1, the virus that causes avian influenza. They found that the extracts, particularly petroleum ether and water extracts, were capable of up to 99.9 % antiviral activity, mainly through the inhibition of virus adsorption into the host cell. More results can be seen in Table 4.

Haslin et al. [133] investigated whether sulfated-cell-wall polysaccharides from the gametic, carposporic and tetrasporic stages of *A. armata* had any in vitro antiviral activity against HIV. They found that, while the carposporic polysaccharides were ineffective, syncytia (i.e., large, multinucleate and, in the case of HIV infection, nonfunctional cells) formation was completely suppressed by the gametic and tetrasporic polysaccharides at 10 μg/mL after the 7th day of infection. Their results suggest that these compounds block the replication of HIV and the formation of syncytia between infected and uninfected cells after the infection. During infection, they inhibit an early step of viral replication, seemingly due to the interaction between the polysaccharides and viral enzymes. The polysaccharides were also found to be non-toxic to host cells. Rhimou et al. [132] found that extracts of *A. armata* had an inhibitory effect on the in vivo replication of the *Herpes simplex* type 1 virus (HSV-1) in Vero cells (African-green-monkey-kidney cell line). The most effective was the water extract, as can be seen in Table 4. Since the antiviral activity was directly proportional to the extract polarity, the results suggest that that the compounds with antiviral activity are mostly polar. The polar extracts were also the least cytotoxic.

These results suggest that both *A. armata* and *A. taxiformis* produce compounds with antiviral activity. Further studies are required to isolate and purify these compounds, as well as to investigate whether these extracts and compounds are effective against other viruses.

### 3.5. Enzyme Inhibition Activity

Enzyme inhibitors are important therapeutic agents. Some famous examples include simvastatin, which is a hydroxy-methylglutaryl-coenzyme-A (HMG-CoA)-reductase inhibitor that decreases low-density-lipoprotein cholesterol (LDL-C), triglycerides and apolipoprotein B and increases high-density-lipoprotein cholesterol [134], sildenafil, which is a cGMP-specific phosphodiesterase-type-5 inhibitor that is commercially sold under the brand name Viagra and used in the treatment of erectile dysfunction [135], and antibiotics such as penicillin and vancomycin that inhibit enzymes involved in bacterial peptidoglycan synthesis [136].

Acetylcholinesterase inhibition is the basis of most approved therapies for Alzheimer’s disease [137], which is the most prevalent neurocognitive disorder in the world and is estimated to be responsible for about two thirds of the dementia cases worldwide [138]. Since current therapeutics have limited potential and serious side effects, researchers have been looking for new therapeutic agents in the natural world, including marine algae. Bettencourt [139] observed that the dichloromethane extracts of *A. taxiformis* inhibited the activity of acetylcholinesterase with an IC_50_ of 116.50 μg/mL, as can be seen in Table 5. Nunes et al. [91] also tested the activity of extracts of *A. taxiformis* against acetylcholinesterase and butyrylcholinesterase. The IC_50_ values obtained are in Table 5. While the positive controls donepezil and galantamine were far more effective at inhibiting acetylcholinesterase, both extracts were more-effective inhibitors of butyrylcholinesterase than donepezil. Custódio et al. [79] reported the inhibitory activity of the *A. armata* methanol extract against acetylcholinesterase as being 58.4% ± 1.0 at a concentration of 10 mg/mL, while the positive control galantamine inhibited the enzyme by 90.3% ± 0.6 at a concentration ten times lower, while the result for butyrylcholinesterase at the same extract concentration was 66.8% ± 1.3, which is much lower than the 80.3% ± 0.1 inhibition by galantamine at 1 mg/mL. It should be noted that 10 mg/mL is an extremely large concentration, too large to be of interest, even in an extract, and also that presenting results as IC_50_ values is more useful than percentages of inhibition when comparing inhibition power.

Using colorimetric assays, Oumaskour et al. [105] found that dichloromethane–methanol (1:1) extracts of *A. armata* could inhibit both elastase and phospholipase A2 (PLA2) (see Table 5). The researchers suggest that, due to this inhibitory activity, *A. armata* metabolites can have an anti-inflammatory effect.

An enzyme whose inhibition is of interest to the cosmetics industry is tyrosinase, which is the main enzyme involved in the synthesis of melanin, a skin-darkening pigment. Therefore, tyrosinase inhibitors are actively pursued for the formulation of skin-bleaching cosmetic products [140]. Only one study was found that investigated the tyrosinase-inhibitory activity of *Asparagopsis* spp. Custódio et al. [79] observed that, at a concentration of 10 mg/mL, the methanol extract of *A. armata* inhibited the activity of tyrosinase by 81.4%. The positive control arbutin was able to inhibit tyrosinase activity by 78.0% at a concentration of 1 mg/mL. Nonetheless, it is worth noting once again that that whatever inhibitor(s) may be present in the extract are probably present at low concentrations.

## 4. Chemical Characterization of *Asparagopsis* Species

As shown in the previous sections, there is significant scientific interest in the bioactivity of *Asparagopsis* extracts obtained by different processes. Unfortunately, the results of these studies have not been accompanied by the corresponding analysis and identification of the active principles present and their respective mechanisms of action.

This does not mean that the chemical constituents of species of this genus have not been studied. This study has been carried out, as we will see in the following points, in a somewhat parallel mode, with few crossing points between these two areas of investigation. However, it is noteworthy that knowledge about the chemical compositions of these species is fundamental to understanding the mechanisms that give them a competitive advantage over other species, as well as to increase their commercial value and, consequently, promote the conservation of biodiversity and ecosystem sustainability.

### 4.1. General Chemical Composition

Several studies were dedicated to determining the nutritional profile of algae of the *Asparagopsis* genus. The most relevant results of these studies are presented in Table 6 and constitute the tip of the iceberg that justifies many of the properties and applications presented by these algae. Table 6 shows that more than 90% of the fresh biomass of *A. armata* and *A. taxiformis* is water [139,145].

Even considering the inter-species differences highlighted in Table 6, the nutritional profile of these two species, in general, fits within what is expected for red algae (See Table 1 of the work published by Salehi et al. [17]).

Polysaccharides are the most abundant constituent in both species, although structural polysaccharides are much more abundant in *A. armata* than in *A. taxiformis* (Table 6). Haslin et al. [147] analyzed the structure and chemical composition of *A. armata*’s hydrosoluble polysaccharides. The polysaccharides were mostly complex galactans, generally consisting of 1,3- and 1,4-linked galactose units reminiscent of agar and carrageenan structures. These galactans have ramifications on C-3 and C-6 and are mostly sulphated on C-4, the latter being a feature of κ-family carrageenans [147].

On the other hand, *A. taxiformis* appears to have a chemical composition richer in lipids, minerals and protein.

Nunes et al. [146] performed a biochemical characterization of seven algae from the Madeira archipelago, including *A. taxiformis*, showing its highest amount of protein, even compared with other red seaweeds, which could, potentially, constitute 31.34 % of the recommended daily protein intake.

In another study, Nunes et al. [91] showed that lipids from *A. taxiformis* are mainly sterols (discussed below), phytols, and fatty-acid derivatives. The saturated fatty acids are the most abundant class (5.65 ± 0.66 mg/g of dry weight), with palmitic acid (C16:0) representing about 72% of the total fatty-acid content. The fatty-acid profile for *A. taxiformis* does not significantly differ from that determined for *A. armata*, with palmitic acid being the most abundant, followed by myristic acid [148]. In both species, the content of polyunsaturated acids is much lower than that of the other fatty acids [91,148]. El-Baroty et al. [149] analyzed the saponifiable lipids of *A. taxiformis* and found a distinct fatty-acid profile, with unsaturated acids as the most abundant class and linolenic acid (C18:3) as the major fatty acid identified. Galindo et al. [150] found an *A. taxiformis* fatty-acid profile where polyunsaturated-fatty-acid content is significant and in which docosahexaenoic acid corresponds to 6.6% of the fatty acids. Different environmental factors and experimental methodologies can justify the reported differences in the fatty-acid profile.

According to Manilal et al. [151], the fractionation of the methanolic extract of the *Falkenbergia* phase of *A. taxiformis* originated an active fraction against human pathogens, whose chemical composition is mostly palmitic and oleic fatty acids. Vedhagiri et al. [122] also isolated a fraction composed mainly of fatty-acid derivatives from *A. taxiformis* which was active against *Leptospira javanica*, which is responsible for leptospirosis disease.

Andrade et al. [80] determined a mannitol content of 34.70 mg/100 g of dry algae, a value corresponding to 47% of the compounds analyzed in this alga by these authors. Considering that mannitol is a natural compound with several industrial and pharmacological applications [152], the ethanolic extract of *A. armata* can be seen as an interesting source of this alcohol sugar.

The biological activities exhibited by the extracts of *Asparagopsis* species and discussed in previous sections, associated with the nutritional profile of these algae, have led to several studies showing the benefits of applying these algae and their extracts as functional ingredients in human and animal foods [150,153,154].

### 4.2. Secondary Metabolites Constituents

Many of the biological activities and potential demonstrated by *Asparagopsis* species are related to their composition in secondary metabolites. A review of the available literature shows that these algae biosynthesize several secondary metabolites such as sterols, but it is the small halogen molecules that are most-often reported and exhibit the greatest chemical diversity in this genus.

#### 4.2.1. Sterols

The ability of phytosterols (i.e., sterols from plant kingdom) to reduce cholesterol levels in humans was first demonstrated in 1953 [155]. Phytosterols reduce total and LDL cholesterol levels in plasma by inhibiting its absorption from the small intestine. Hence, they lower the risk of atherosclerosis and offer protection against cardiovascular diseases [156]. They are also known to reduce the risk of some cancers, such as breast, prostate and colon cancer [157] and to have anti-inflammatory and immunomodulatory properties [158].

*Asparagopsis* species are a source of sterols. El Hattab et al. [159] used FTIR (Fourier-transform infrared spectrometry) and HPLC (high-performance liquid chromatography) to determine the total amount of sterols in *A. armata*. The percentages obtained were 3.37% (*w*/*w*) using FTIR and 3.30% (*w*/*w*) using HPLC, demonstrating a high degree of agreement between the two methods of analysis. A careful analysis of this work does not provide clarification of whether the unit % (*w*/*w*) presented corresponds to % by weight of the seaweed or extract. Lopes et al. [160] studied the sterol profiles of 18 green, brown and red macroalgae from the Portuguese coast, including *A. armata*, which was the species with the lowest content of sterols (55.5 mg/100 g of dry biomass). Unfortunately, based on these two studies, it is not possible to compare the sterol contents as the units presented are not directly comparable.

**Figure 1:**
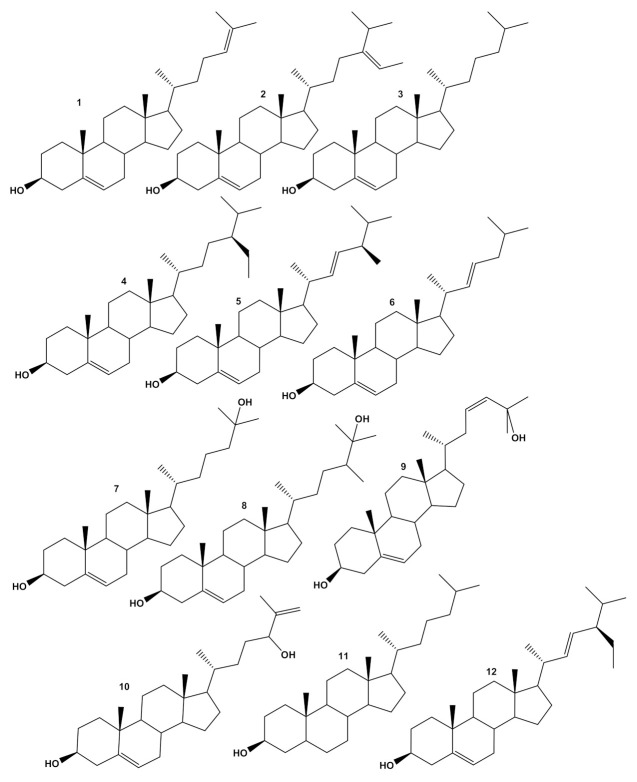
Chemical structures of sterols identified in *Asparagospis* species.

The first research about the sterol profile of *A. armata* identified desmosterol (**1**), fucosterol (**2**), cholesterol (**3**), β-sitosterol (**4**), brassicasterol (**5**), 22-dehydro-cholesterol (**6**), 25-hydroxy-cholesterol (**7**), 25-hydroxy-24-methylcholesterol (**8**), cholesta-5,23-diene-3β,25-diol (known as liagosterol) (**9**), and cholesta-5,25-dien-3β,24-diol (**10**) (Figure 1) [161]. These authors [162] further clarify that compounds **9** and **10** were identified in fresh algae, resulting from the natural oxidation of desmosterol (**1**), and not just artifacts caused by reactions during the processing of the algae sample. Using HPLC, Lopes et al. [160] determined the content of desmosterol (**1**), fucosterol (**2**), cholesterol (**3**) and β-sitosterol (**4**) (Figure 1) as being 71.1, 174.3, 289.2 and 20.4 mg/100 g of *A. armata* dry biomass, respectively. In the same species, Andrade et al. [74] identified, using GC-MS, cholestanol (**11**) at 17.70 mg/ 100 g of dry biomass, while β-sitosterol and fucosterol were not detected.

As has been known since 1979 [161], the sterol composition of *A. armata* significantly depends on the stage of its life cycle, differing between the gametophyte and tetrasporophyte. Thus, it is very relevant that the authors indicate in their studies the life-cycle stage of the alga under study.

It is surprising that the sterol profile of *A. taxiformis* is not described in the literature. Only Nunes et al. [91] suggest the presence of cholesterol (**3**) in this species based on a qualitative analysis by TLC, while El-Baroty et al. [149], besides cholesterol (**3**), suggest the presence of stigmasterol (**12**) in the unsaponifiable lipids of *A. taxiformis*.

#### 4.2.2. Halogenated Compounds

A significant number and diversity of halogenated metabolites have been identified in red algae, including *Asparagopsis* species [163].

The first detailed studies on halogenated compounds biosynthesized by *Asparagospsis* species were published between 1974 and 1979 and concern the study of essential oils and the volatile fraction of *A. taxiformis* [65,66,67,68,164,165]. It was only at the end of this decade that studies involving *A. armata* appeared [68]. These halogenated compounds are the most volatile, with low molecular weights, one-to-four-carbon chains, one or more halogen atoms, and other functional groups such as ketones, alcohols, carboxylic acids, and unsaturated bonds.

Kladi et al. [163] elaborated a very complete and clear table (see Table 3 in the cited reference) with all the halogenated volatile compounds (C1–C10) and chemical structures identified in *Asparagopsis* species until 2003. Additionally, very recently, Félix et al. [145] published a review of *A. armata* in which they also present a table with the halogenated compounds already identified in this species.

**Figure 2:**
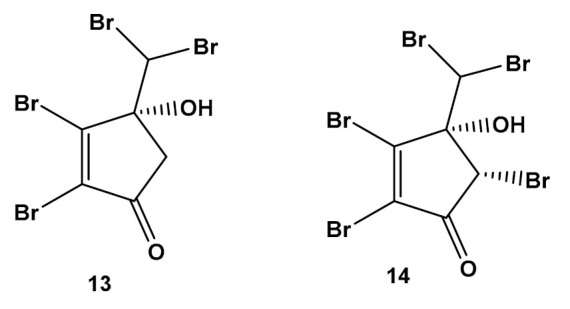
Chemical structures of two 2,3-dibromocyclopentone derivatives identified in *Asparagospis taxiformis*.

Few studies were published in the last few decades concerning new halogenated secondary metabolites. Greff et al. [69] conducted phytochemical research on *A. taxiformis* that revealed the existence of two new, highly brominated cyclopentones: mahorone (**13**) and 5-bromomahorone (**14**) (Figure 2), which are the first two documented examples of natural derivatives of 2,3-dibromocyclopentone.

The high toxicity of compounds **13** and **14** against a marine pathogen [69] suggests that the compounds are relevant to the interaction with other marine species and to the competitive advantage exhibited by *A. taxiformis*. The pharmacological potential of these compounds was discussed in a previous section.

The interest in the biological and ecological role of halogenated secondary metabolites in *Asparagopsis* species has led to the publication of several works whose main conclusions are discussed below.

Of note is the work of Marshall et al. [166] in which the volatile halocarbons produced by *A. taxiformis* and *A. armata* tetrasporophytes from several geographically distant areas were determinated. Bromoform, dibromomethane, dibromochloromethane, 1,2-dibromoethylene and tribromoethylene were identified as algal metabolites. These researchers also found that there are quantitative and qualitative differences between the various samples regarding the release of halocarbons and that increasing the intensity of the light that algae are exposed to results in an increased halocarbon release. The released amount varied between 8 and 866 ng/g of fresh weight for bromoform, 3 and 59 ng/g of fresh weight for dibromomethane and 0.4 and 17 ng/g of fresh weight for 1,2-dibromoethylene.

The same team [167] shed light on the metabolism of halogenated compounds, on the plant organs involved in the accumulation and release of these compounds, and on the abiotic factors that influence these processes. Papers by Paul et al. [100,168] show that the most-abundant halogenated metabolites of the *A. armata* methanol extract are bromoform (on average of 1.45% and 1.67% of the dry weight of the tetrasporophyte and the gametophyte, respectively) and dibromoacetic acid (average values of 0.74% and 0.25% of dry weight of the tetrasporophyte and the gametophyte, respectively), and the release of halogenated metabolites to the surface and surrounding seawater is ecologically relevant. They seem to be responsible for the activity against epiphytic bacteria, contributing to the defense mechanism of *A. armata*. Paul et al. [100] also identified bromochloroacetic acid and dibromochloromethane (at 0.14% and 0.08%, 0.02% and 0.03% dry weight on the tetrasporophyte and the gametophyte, respectively) and dibromoacrylic acid in the *A. armata* methanol extract.

The anti-methanogenic activity of *Asparagopsis* species is described in the literature, and a very significant effect was observed with the incorporation of a small % of these algae into in vivo systems, without any short-term toxic effects [169,170]. The halomethane-type compounds are most often responsible for the effect of inhibiting ruminant microbial methanogenesis [171]. Taking into account that the main compound responsible for this activity seems to be bromoform, a standardized protocol for the quantification of this secondary metabolite in biomass was developed by Romanazzi et al. [172]. However, it cannot be ruled out that other secondary metabolites, such as tannins and flavonoids, may exert this anti-methanogenic effect [173]. Concerning the use of *Asparagopsis* spp. in animal feed, some caution is recommended, since Li et al. [174] identified granulomatous and keratotic ruminal-mucosa changes in sheep supplemented with *Asparagopsis*, while Muizelaar et al. [175] detected bromoform in the milk of lactating cows that had *Asparagopsis* incorporated into their feed, and found abnormalities in their rumen wall, with visible signs of inflammation. It should be noted that Silva et al. [176] reported oxidative stress and neurotoxicity in the shrimp *Palaemon elegans*.

Knowing that *A. armata* has compounds that have a significant impact on the development of other species in the invaded ecosystem, the exudate of this alga was tested as an herbicide [177]. The results showed that in addition to being effective, the exudate shows a mechanism of action similar to that exhibited by classic chemical biocides.

#### 4.2.3. Other Compounds

Broadgate et al. [178] demonstrated for the first time that macroalgae release isoprene and other non-methane hydrocarbons (NMHCs). NMHCs are important in controlling the balance of atmospheric oxidants and altering air quality on both local and global scales by perturbing levels of nitrogen oxides and ozone in the troposphere [179,180]. The rate of production of several NMHCs by *A. armata*, in pmol/g of dry weight/hour, was as follows: ethene—39.92; *n*-pentane—12.88; ethane—11.35; propene—10.43; propane—5.29; isoprene—5.26 [178]. These researchers also observed that emissions are temperature dependent and related to light availability.

**Figure 3:**
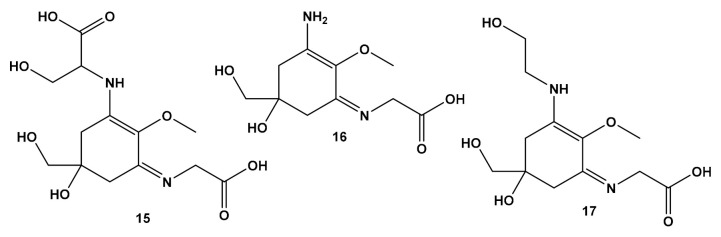
Chemical structure of mycosporine-like amino acids accumulated in *A. armata*.

Being aware of the high nitrogen removal rates of *A. armata* biofilters [8], Figueroa et al. [181] used the tank-cultivated tetrasporophyte of *A. armata* as a system to analyze the accumulation of mycosporine-like amino acids (MAAs) in the seaweed. The cosmetics industry is interested in MAAs because these molecules absorb ultraviolet radiation, which means they can be used as ingredients in sunscreens [182]. Figueroa et al. [181] observed that the concentration of MAAs increases with total ammonia and nitrogen (TAN) fluxes lower than 100 µM/h. Three MAAs were identified (Figure 3): shinorine (**15**) (80–85%), palythine (**16**) (17–20%) and asterina-330 (**17**) to be present only at residual concentrations. The authors [181] highlighted the potential of *A. armata* to produce significant amounts of commercially valuable MAAs while performing as a biofilter.

Nunes et al. [146] quantified some compounds with known antioxidant activity. The results obtained for 100 g of dry weight were as follows: chlorophyll a—28.21 mg; carotenoids—13.14 mg; total phenolic compounds (TPCs)—57.63 mg gallic-acid equivalent/100 g dry biomass; total flavonoid content (TFC)—19.26 mg quercetin equivalent/100 g dry biomass [140], while Sahnouni et al. [183] obtained the values of 14.11 ± 0.02 mg gallic-acid equivalent/g extract and 8.86 ± 0.006 mg quercetin equivalent/g extract to TPC and TFC, respectively. These values are not comparable since they are expressed in non-equivalent units.

Although previous studies have quantified the total content of phenolic compounds and flavonoids, the unambiguous structures of the quantified metabolites have not been elucidated so far. This is an open point that must be investigated and clarified, as flavonoids and other phenolic compounds are very interesting from the point of view of their biological activities, applications, and as scaffolds for added-value compounds.

## 5. Conclusions

There is no doubt that, although the genus *Asparagopsis* includes only two species whose taxonomic classification is widely accepted, this is a genus that raises a lot of scientific curiosity. The scientific interest encompasses diverse and complementary fields. They range from populational studies to the development of commercial applications, through studies of their biology, ecology, bioactivities, and chemical composition. *Asparagopsis* species are considered invasive in various parts of the world, and there is a large amount of biomass that has no commercial value and therefore needs to be valued.

From this literature review, it is concluded that extracts obtained by different techniques and different solvents have been the most studied. The scientific community has been mainly dedicated to the evaluation of the activity of these extracts against different microorganisms, as cytotoxic agents, antioxidants, antimethanogenics, as well as enzyme inhibitors. However, an analysis of the published results shows that very different methodologies are applied and, often, the results are not comparable. Additionally, the activity level displayed is generally found to be low when compared to a suitable positive control.

A significant conclusion that can be drawn from the literature reviewed above is this: compared to the large number of studies on the biological activity of algae extracts of the genus *Asparagopsis*, very little is known about the compounds responsible for the observed effects. There are few published works in which the chemical compositions of the evaluated extracts were analyzed, or the pure compounds to which the observed biological effects are potentially associated were tested.

Regarding the constituents of *Asparagopsis* extracts, the fatty-acid and sterol compositions as well as volatile halogenated compounds are the best known. The researchers attribute many biological activities to these compounds, even though they have rarely been tested in an isolated and purified form.

Several studies suggest the presence of phenolic compounds, such as flavonoids, in extracts from these species. However, to date, no secondary metabolite of this family has been isolated and identified in these species. In fact, very little is known about the polar *Asparagopsis* secondary metabolites.

Considering the environmental effects and toxicity of halogenated compounds against invertebrates and vertebrates suggested in several studies, the application of these algae in human and animal food should be considered with caution.

Thus, despite the many studies published and reviewed here, it seems clear that there is still a long way to go before an economically viable, effective, and safe application from an environmental and human point of view can be suggested.

## Figures and Tables

**Table 1:** Antioxidant activity of *Asparagopsis* extracts.

Species	Experimental Assay	Extract	Result	Units	Ref.
*A. armata*	DPPH	Dichloromethane-mehtanol 1:1	6.25	EC_50_ mg/mL	[77]
		BHA (positive control)	0.04	EC_50_ mg/mL	[77]
		BHT (positive control)	0.06	EC_50_ mg/mL	[77]
		Ascorbic acid (positive control)	0.06	EC_50_ mg/mL	[77]
		α-tocopherol (positive control)	0.014	EC_50_ mg/mL	[77]
		Methanol	0.86	EC_50_ mg/mL	[78]
		BHA (positive control)	0.008	EC_50_ mg/mL	[78]
		BHT (positive control)	0.011	EC_50_ mg/mL	[78]
		Methanol	23.9 ± 0.3	% radical-scavenging activity at 10 mg/mL	[79]
	Nitric oxide	Ethanol	14.33	EC_25_ mg dry algae/mL	[80]
	Deoxyribose test	Water	68.76	% inhibition at 1 mg/mL	[78]
*A. taxiformis*	Hydrogen-peroxide scavenging	Chloroform	11.07 ± 0.151	% inhibition at 500 µg/mL	[81]
		Petroleum ether	10.88 ± 0.139	% inhibition at 500 µg/mL	[81]
		Methanol	10.77 ± 0.131	% inhibition at 500 µg/mL	[81]
		Ethyl acetate	10.25 ± 0.136	% inhibition at 500 µg/mL	[81]
		Ascorbic acid (positive control)	17.59 ± 0.222	% inhibition at 500 µg/mL	[81]
	Superoxide-radical scavenging	Methanol	85.00 ± 0.002	% scavenging activity at 500 µg/mL	[81]
		Chloroform	79.21 ± 0.006	% scavenging activity at 500 µg/mL	[81]
		Petroleum ether	47.00 ± 0.018	% scavenging activity at 500 µg/mL	[81]
		Ethyl acetate	45.00 ± 0.008	% scavenging activity at 500 µg/mL	[81]
		Ascorbic acid (positive control)	87.11 ± 0.0005	% scavenging activity at 500 µg/mL	[81]
	FRAP (ferric-reducing antioxidant power)	Chloroform	67.19 ± 0.0005	% antioxidant activity at 500 µg/mL	[81]
		Methanol	65.63 ± 0.001	% antioxidant activity at 500 µg/mL	[81]
		Petroleum ether	64.06 ± 0.0005	% antioxidant activity at 500 µg/mL	[81]
		Ethyl acetate	54.69 ± 0.0005	% antioxidant activity at 500 µg/mL	[81]
		Ascorbic acid (positive control)	73.44 ± 0.002	% antioxidant activity at 500 µg/mL	[81]
	Reducing activity	Water (M1)	233.15 ± 5.15	mg AAE/100 g dw ^a^	[82]
		Water (M2)	174.38 ± 11.65	mg AAE/100 g dw	[82]
		Ethanol (M1)	1908.44 ± 59.15	mg AAE/100 g dw	[82]
		Ethanol (M2)	1156.86 ± 13.87	mg AAE/100 g dw	[82]
		Methanol (M1)	584.46 ± 15.36	mg AAE/100 g dw	[82]
		Methanol (M2)	1161.47 ± 14.43	mg AAE/100 g dw	[82]
		Ethyl acetate (M1)	409.60 ± 10.84	mg AAE/100 g dw	[82]
		Ethyl acetate (M2)	707.42 ± 98.78	mg AAE/100 g dw	[82]
	DPPH	Water (M1)	4.65 ± 0.29	IC_50_ mg/mL	[82]
		Water (M2)	1.37 ± 0.03	IC_50_ mg/mL	[82]
		Ethanol (M1)	1.54 ± 0.07	IC_50_ mg/mL	[82]
		Ethanol (M2)	1.37 ± 0.04	IC_50_ mg/mL	[82]
		Methanol (M1)	2.69 ± 0.03	IC_50_ mg/mL	[82]
		Methanol (M2)	1.64 ± 0.01	IC_50_ mg/mL	[82]
		Ethyl acetate (M1)	3.62 ± 0.04	IC_50_ mg/mL	[82]
		Ethyl acetate (M2)	1.44 ± 0.08	IC_50_ mg/mL	[82]
	Ferrous-ion chelation	Water (M1)	113.01 ± 10.62	IC_50_ mg/mL	[82]
		Water (M2)	74.00 ± 1.81	IC_50_ mg/mL	[82]
		Ethanol (M1)	5.26 ± 0.27	IC_50_ mg/mL	[82]
		Ethanol (M2)	10.49 ± 0.44	IC_50_ mg/mL	[82]
		Methanol (M1)	8.36 ± 0.29	IC_50_ mg/mL	[82]
		Methanol (M2)	10.07 ± 0.18	IC_50_ mg/mL	[82]
		Ethyl acetate (M1)	1.57 ± 0.03	IC_50_ mg/mL	[82]
		Ethyl acetate (M2)	5.88 ± 0.26	IC_50_ mg/mL	[76]

M1—Extraction by sonication + stirring; M2—Sohxlet extraction; ^a^ mg l-ascorbic acid equivalent per 100 g of dry weight.

**Table 2:** Anticancer activity of *Asparagopsis* spp.

Species	Experimental Assay	Extract/Control	Result	Units	Ref.
*A. armata*	Cytotoxicity (Daudi cells)	Dichloromethane–ethanol (1:1)	32.52 ± 7.33	% reduction of viable cells at 100 μg/mL after 24 h incubation	[77]
	Cytotoxicity (Jurkat cells)		30.07 ± 7.24	% reduction of viable cells at 100 μg/mL after 24 h incubation	[77]
	Cytotoxicity (HepG-2 cells)	Methanol	567.9	IC_50_ in μg/mL	[89]
		Dichloromethane	473.1	IC_50_ in μg/mL	[89]
		Cisplatin (positive control)	136.5	IC_50_ in μg/mL	[89]
	Cytotoxicity (Caco-2 cells)	Methanol	823.0	IC_50_ in μg/mL	[89]
		Dichloromethane	531.6	IC_50_ in μg/mL	[89]
		Cisplatin (positive control)	80.11	IC_50_ in μg/mL	[89]
	Antiproliferative activity (HepG-2 cells)	Methanol	857.3	IC_50_ in μg/mL	[89]
		Dichloromethane	518.9	IC_50_ in μg/mL	[89]
		Cisplatin (positive control)	22.63	IC_50_ in μg/mL	[89]
		Tamoxifen (positive control)	16.97	IC_50_ in μg/mL	[89]
	Antiproliferative activity (Caco-2 cells)	Methanol	508.1	IC_50_ in μg/mL	[90]
		Dichloromethane	271.5	IC_50_ in μg/mL	[90]
		Cisplatin (positive control)	92.00	IC_50_ in μg/mL	[90]
*A. taxiformis*	Cytotoxicity (A549 cells)	Chloroform-methanol (2:1)	98.02 ± 0.23	IC_50_ in μg/mL	[91]
		Ethanol	>200	IC_50_ in μg/mL	[91]
		Colchicine (positive control)	2.78 ± 0.71	IC_50_ in μg/mL	[91]

**Table 3:** Antimicrobial activity of *Asparagopsis* extracts.

Species	Experimental Assay	Extract/Compound	Target Species	Result	Units	Ref.
*A. armata*	Agar-diffusion method	Dichloromethane (2 mg/disk)	*Vibrio anguillarum*	19.3 ± 1.3	Inhibition zone diameter (mm)	[101]
			*Pseudomonas anguilliseptica*	26.9 ± 2.2	Inhibition zone diameter (mm)	[101]
			*Aeromonas salmonicida*	17.0 ± 2.2	Inhibition zone diameter (mm)	[101]
			*Aeromonas hydrophila*	14.9 ± 2.7	Inhibition zone diameter (mm)	[101]
			*Yersinia ruckeri*	15.3 ± 1.7	Inhibition zone diameter (mm)	[101]
		Solid (fresh) (2 mg/well)	*Bacillus subtilis*	29.4	Inhibition zone diameter (mm)	[102]
			*Bacillus cereus*	30.2	Inhibition zone diameter (mm)	[102]
			*Staphylococcus aureus*	22.2	Inhibition zone diameter (mm)	[102]
			*Escherichia coli*	20.8	Inhibition zone diameter (mm)	[102]
			*Pseudomonas aeruginosa*	25.5	Inhibition zone diameter (mm)	[102]
			*Candida albicans*	32	Inhibition zone diameter (mm)	[102]
		Solid (lyophilized) (2 mg/well)	*Bacillus subtilis*	38.9	Inhibition zone diameter (mm)	[102]
			*Bacillus cereus*	51.1	Inhibition zone diameter (mm)	[102]
			*Staphylococcus aureus*	35.1	Inhibition zone diameter (mm)	[102]
			*Escherichia coli*	39.9	Inhibition zone diameter (mm)	[102]
			*Pseudomonas aeruginosa*	27.3	Inhibition zone diameter (mm)	[102]
			*Candida albicans*	53.2	Inhibition zone diameter (mm)	[102]
		Methanol (0.3 mg/disk)	*Bacillus subtilis*	11	Inhibition zone diameter (mm)	[93]
		Dichloromethane (0.3 mg/disk)	*Bacillus subtilis*	12	Inhibition zone diameter (mm)	[93]
		*n*-Hexane (0.3 mg/disk)	*Bacillus subtilis*	8	Inhibition zone diameter (mm)	[93]
		Chloramphenicol	*Bacillus subtilis*	30	Inhibition zone diameter (mm)	[93]
	Fungal growth measurement	Dichloromethane	*Saccharomyces cerevisiae*	119.8	IC_50_ in μg/mL	[93]
		*n*-hexane	*Saccharomyces cerevisiae*	97.6	IC_50_ in μg/mL	[93]
		Amphotericin B (positive control)	*Saccharomyces cerevisiae*	21.6	IC_50_ in μg/mL	[93]
	Broth microdilution	Dichloromethane—methanol (1:1)	*Bacillus subtilis*	83.7	IC_50_ in μg/mL	[103]
			*Escherichia coli*	540.6	IC_50_ in μg/mL	[103]
			*Pseudomonas aeruginosa*	374.3	IC_50_ in μg/mL	[103]
			*Salmonella enteritidis*	613.8	IC_50_ in μg/mL	[103]
			*Staphylococcus aureus*	528.6	IC_50_ in μg/mL	[103]
	Agar-diffusion method (using 3 cm thallus)	Fresh thallus	*Bacillus subtilis*	11.0	Inhibition zone diameter (mm)	[104]
			*Escherichia coli*	9.0	Inhibition zone diameter (mm)	[104]
			*Morganella morganii*	11.0	Inhibition zone diameter (mm)	[104]
			*Staphylococcus aureus*	12.0	Inhibition zone diameter (mm)	[104]
			*Streptococcus pyogenes*	12.0	Inhibition zone diameter (mm)	[104]
	Agar-diffusion method	Methanol (500 μg/disk)	*Bacillus cereus*	10–15	Inhibition zone diameter (mm)	[105]
			*Clostridium sporogenes*	10–15	Inhibition zone diameter (mm)	[105]
		Acetone	*Staphylococcus aureus ssp aureus*	>15	Inhibition zone diameter (mm)	[105]
		Chloroform	*Staphylococcus aureus ssp aureus*	10–15	Inhibition zone diameter (mm)	[105]
		Dichloromethane—methanol (1:1)	*Bacillus cereus*	10–15	Inhibition zone diameter (mm)	[105]
	Bacterial growth measurement	Dichloromethane—methanol (1:1)	*Aeromonas hydrophila*	5.7 ± 0.1	% growth inhibition at 1 mg/mL	[106]
		Dichloromethane—methanol (1:1) Fraction 1 by VLC	*Aeromonas hydrophila*	836.1	IC_50_ in μg/mL	[106]
		Dichloromethane—methanol (1:1) Fraction 2 by VLC	*Aeromonas hydrophila*	293.5	IC_50_ in μg/mL	[106]
		Dichloromethane—methanol (1:1) Fraction 3 by VLC	*Aeromonas hydrophila*	162.9	IC_50_ in μg/mL	[106]
		Dichloromethane—methanol (1:1) Fraction 4 by VLC	*Aeromonas hydrophila*	191.9	IC_50_ in μg/mL	[106]
		Dichloromethane—methanol (1:1) Fraction 5 by VLC	*Aeromonas hydrophila*	265.1	IC_50_ in μg/mL	[106]
		Dichloromethane—methanol (1:1) Fraction 6 by VLC	*Aeromonas hydrophila*	379.0	IC_50_ in μg/mL	[106]
		Dichloromethane—methanol (1:1)	*Aeromonas aquariorum*	23.5 ± 0.6	% growth inhibition at 1 mg/mL	[106]
		Dichloromethane—methanol (1:1) Fraction 1 by VLC	*Aeromonas aquariorum*	>1000	IC_50_ in μg/mL	[106]
		Dichloromethane—methanol (1:1) Fraction 2 by VLC	*Aeromonas aquariorum*	588.1	IC_50_ in μg/mL	[106]
		Dichloromethane—methanol (1:1) Fraction 3 by VLC	*Aeromonas aquariorum*	443.8	IC_50_ in μg/mL	[106]
		Dichloromethane—methanol (1:1) Fraction 4 by VLC	*Aeromonas aquariorum*	437	IC_50_ in μg/mL	[106]
		Dichloromethane—methanol (1:1) Fraction 5 by VLC	*Aeromonas aquariorum*	421.1	IC_50_ in μg/mL	[106]
		Dichloromethane—methanol (1:1) Fraction 6 by VLC	*Aeromonas aquariorum*	872.4	IC_50_ in μg/mL	[106]
		Dichloromethane—methanol (1:1)	*Edwardsiella tarda*	86.0 ± 8.3	% growth inhibition at 1 mg/mL	[106]
		Dichloromethane—methanol (1:1) Fraction 1 by VLC	*Edwardsiella tarda*	414.1	IC_50_ in μg/mL	[106]
		Dichloromethane—methanol (1:1) Fraction 2 by VLC	*Edwardsiella tarda*	152.7	IC_50_ in μg/mL	[106]
		Dichloromethane—methanol (1:1) Fraction 3 by VLC	*Edwardsiella tarda*	54.4	IC_50_ in μg/mL	[106]
		Dichloromethane—methanol (1:1) Fraction 4 by VLC	*Edwardsiella tarda*	75.1	IC_50_ in μg/mL	[106]
		Dichloromethane—methanol (1:1) Fraction 5 by VLC	*Edwardsiella tarda*	83.8	IC_50_ in μg/mL	[106]
		Dichloromethane—methanol (1:1) Fraction 6 by VLC	*Edwardsiella tarda*	244.5	IC_50_ in μg/mL	[106]
		Dichloromethane—methanol (1:1)	*Vibrio anguillarum*	35.1 ± 5.7	% growth inhibition at 1 mg/mL	[106]
		Dichloromethane—methanol (1:1) Fraction 1 by VLC	*Vibrio anguillarum*	41.1	IC_50_ in μg/mL	[106]
		Dichloromethane—methanol (1:1) Fraction 2 by VLC	*Vibrio anguillarum*	19.6	IC_50_ in μg/mL	[106]
		Dichloromethane—methanol (1:1) Fraction 3 by VLC	*Vibrio anguillarum*	41.7	IC_50_ in μg/mL	[106]
		Dichloromethane—methanol (1:1) Fraction 4 by VLC	*Vibrio anguillarum*	60.4	IC_50_ in μg/mL	[106]
		Dichloromethane—methanol (1:1) Fraction 5 by VLC	*Vibrio anguillarum*	52.5	IC_50_ in μg/mL	[106]
		Dichloromethane—methanol (1:1) Fraction 6 by VLC	*Vibrio anguillarum*	89.9	IC_50_ in μg/mL	[106]
		Dichloromethane—methanol (1:1)	*Photobacterium damselae*	0.0 ± 4.1	% growth inhibition at 1 mg/mL	[106]
		Dichloromethane—methanol (1:1) Fraction 1 by VLC	*Photobacterium damselae*	18.9	IC_50_ in μg/mL	[106]
		Dichloromethane—methanol (1:1) Fraction 2 by VLC	*Photobacterium damselae*	14.5	IC_50_ in μg/mL	[106]
	Bacterial growth measurement	Dichloromethane—methanol (1:1) Fraction 3 by VLC	*Photobacterium damselae*	13.6	IC_50_ in μg/mL	[106]
		Dichloromethane—methanol (1:1) Fraction 4 by VLC	*Photobacterium damselae*	20.0	IC_50_ in μg/mL	[106]
		Dichloromethane—methanol (1:1) Fraction 5 by VLC	*Photobacterium damselae*	11.7	IC_50_ in μg/mL	[106]
		Dichloromethane—methanol (1:1) Fraction 6 by VLC	*Photobacterium damselae*	17.3	IC_50_ in μg/mL	[106]
	Alamar-blue assay [101]	Hexane	*Leishmania donovani*	>40	IC_50_ in μg/mL	[107]
		Dichloromethane	*Leishmania donovani*	>40	IC_50_ in μg/mL	[107]
		Ethanol (VLC fraction eluted with hexane—ethyl acetate (1:1))	*Leishmania donovani*	10	IC_50_ in μg/mL	[107]
		Ethanol (VLC fraction eluted with ethyl acetate)	*Leishmania donovani*	19	IC_50_ in μg/mL	[107]
		Pentamidin (positive control)	*Leishmania donovani*	0.9–1	IC_50_ in μg/mL	[107]
		Amphotericin B (positive control)	*Leishmania donovani*	0.18–0.19	IC_50_ in μg/mL	[107]
*A. taxiformis*	Antibacterial/antifungal activity (growth measurement)	Mahorone	*Acinetobacter baumannii*	8	MIC_80_ in μg/mL	[69]
			*Escherichia coli* (2884)	>32	MIC_80_ in μg/mL	[69]
			*Escherichia coli* (5746)	16	MIC_80_ in μg/mL	[69]
			*Pseudomonas aeruginosa*	>10	MIC_80_ in μg/mL	[69]
			*Staphylococcus aureus* (MRSA)	16	MIC_80_ in μg/mL	[69]
			*Staphylococcus aureus* (MSSA)	>32	MIC_80_ in μg/mL	[69]
			*Aspergillus fumigatus*	>32	MIC_80_ in μg/mL	[69]
			*Candida albicans*	>32	MIC_80_ in μg/mL	[69]
		5-bromomahorone	*Acinetobacter baumannii*	16	MIC_80_ in μg/mL	[69]
			*Escherichia coli* (2884)	>32	MIC_80_ in μg/mL	[69]
			*Escherichia coli* (5746)	32	MIC_80_ in μg/mL	[69]
			*Pseudomonas aeruginosa*	>10	MIC_80_ in μg/mL	[69]
			*Staphylococcus aureus* (MRSA)	16	MIC_80_ in μg/mL	[69]
			*Staphylococcus aureus* (MSSA)	>32	MIC_80_ in μg/mL	[69]
			*Aspergillus fumigatus*	>32	MIC_80_ in μg/mL	[69]
			*Candida albicans*	>32	MIC_80_ in μg/mL	[69]
		Rifampicin (positive control)	*Acinetobacter baumannii*	1	MIC_80_ in μg/mL	[69]
		Novobiocin (positive control)	*Escherichia coli* (2884)	0.3	MIC_80_ in μg/mL	[69]
		Novobiocin (positive control)	*Escherichia coli* (5746)	0.1	MIC_80_ in μg/mL	[69]
		Ciprofloxacin (positive control)	*Pseudomonas aeruginosa*	1.5	MIC_80_ in μg/mL	[69]
		Imipenem (positive control)	*Staphylococcus aureus* (MRSA)	8	MIC_80_ in μg/mL	[69]
		Penicillin (positive control)	*Staphylococcus aureus* (MSSA)	0.01	MIC_80_ in μg/mL	[69]
	Alamar-blue assay [108]	Hexane	*Leishmania donovani*	17	IC_50_ in μg/mL	[107]
		Dichloromethane	*Leishmania donovani*	16	IC_50_ in μg/mL	[107]
		Ethanol (VLC fraction eluted with hexane–ethyl acetate (1:1))	*Leishmania donovani*	14	IC_50_ in μg/mL	[107]
		Ethanol (VLC fraction eluted with ethyl acetate)	*Leishmania donovani*	20	IC_50_ in μg/mL	[107]

**Table 4:** Antiviral activity of *Asparagopsis* extracts.

Species	Experimental Assay	Extract	Target Virus	Result	Units	Ref.
*A. taxiformis*	Plaque infectivity reduction	Hexane (20 μg/mL)	H5N1	50	% inhibition	[131]
		Hexane (40 μg/mL)	H5N1	57	% inhibition	[131]
		Petroleum ether (20 μg/mL)	H5N1	73	% inhibition	[131]
		Petroleum ether (40 μg/mL)	H5N1	>99.9	% inhibition	[131]
		Ethyl acetate (20 μg/mL)	H5N1	46	% inhibition	[131]
		Ethyl acetate (40 μg/mL)	H5N1	55	% inhibition	[131]
		Methylene chloride–ethanol (1:1) (20 μg/mL)	H5N1	0	% inhibition	[131]
		Methylene chloride–ethanol (1:1) (40 μg/mL)	H5N1	15	% inhibition	[131]
		Water (20 μg/mL)	H5N1	>99.9	% inhibition	[131]
		Water (40 μg/mL)	H5N1	>99.9	% inhibition	[131]
*A. armata*	Antiviral assays based on cell viability	Methanol	HSV-1	7.7	EC_50_ in μg/mL	[132]
		Choloform–methanol (3:2)	HSV-1	40.9	EC_50_ in μg/mL	[132]
		Dichloromethane	HSV-1	22.8	EC_50_ in μg/mL	[132]
		Water	HSV-1	<2.5	EC_50_ in μg/mL	[132]

**Table 5:** Enzyme inhibition activity of *Asparagopsis* spp.

Species	Target Enzyme	Experimental Assay	Extract/Compound	Result	Units	Ref.
*A. armata*	Acetylcholinesterase	Ellman method [141]	Methanol	58.4 ± 1.0	% inhibition at 10 mg/mL	[79]
			Galantamine (positive control)	90.3 ± 0.6	% inhibition at 1 mg/mL	[79]
	Butyrylcholinesterase	Ellman method [141]	Methanol	66.8 ± 1.3	% inhibition at 10 mg/mL	[79]
			Galantamine (positive control)	80.3 ± 0.1	% inhibition at 1 mg/mL	[79]
	Tyrosinase	Method reported by Nerya et al. [142]	Methanol	81.4 ± 5.2	% inhibition at 10 mg/mL	[79]
			Arbutin (positive control)	78.0 ± 0.1	% inhibition at 1 mg/mL	[79]
	Phospholipase A2	Colorimetric assay [143]	Dichloromethane-methanol (1:1)	100	% inhibition at 1 μg/mL	[105]
	Elastase	Colorimetric assay [144]	Dichloromethane-methanol (1:1)	55	% inhibition at 10 μg/mL	[105]
*A. taxiformis*	Acetylcholinesterase	Ellman method [141]	Dichloromethane	116.50 ± 10.94	IC_50_ in μg/mL	[139]
			Chloroform-methanol (2:1)	8.92 ± 0.43	IC_50_ in μg/mL	[91]
			Ethanol	46.33 ± 6.02	IC_50_ in μg/mL	[91]
			Donepezil (positive control)	0.01 ± 0.00	IC_50_ in μg/mL	[91]
			Galantamine (positive control)	0.43 ± 0.09	IC_50_ in μg/mL	[91]
	Butyrylcholinesterase	Ellman method [141]	Chloroform-methanol (2:1)	13.96 ± 0.32	IC_50_ in μg/mL	[91]
			Ethanol	28.10 ± 0.93	IC_50_ in μg/mL	[91]
			Donepezil (positive control)	55.62 ± 3.47	IC_50_ in μg/mL	[91]
			Galantamine (positive control)	>150	IC_50_ in μg/mL	[91]

The results of these studies should encourage further research on this topic.

**Table 6:** General composition of *Asparagopsis* species.

Constituents	*A. armata*	*A. taxiformis*
Water	90.8–91.2 g/100 g fw [139,145]	92.6 g/100 g fw [139]
Polysaccharides	Starch 1.26 g/100 g dw [145]	Starch 8.03 ± 0.38 g/100 g dw [146]
	Other 72.0 g/100 g dw [145]	Other 32.47 ± 1.04 g/100 g dw [146]
Protein	10.9–14.0 g/100 g dw [145]	17.55 ± 0.11 g/100 g dw [146]
Lipids	2.51 ± 0.26 g/100 g dw [145]	6.62 ± 0.54 g/100 g dw [146]
Ashes	13.36 ± 0.68 g/100 g dw [145]	23.76 ± 0.48 g/100 g dw [146]

fw—fresh-weight basis; dw—dry-weight basis.

## Data Availability

Data sharing not applicable.
